# Classifying cells with Scasat, a single-cell ATAC-seq analysis tool

**DOI:** 10.1093/nar/gky950

**Published:** 2018-10-18

**Authors:** Syed Murtuza Baker, Connor Rogerson, Andrew Hayes, Andrew D Sharrocks, Magnus Rattray

**Affiliations:** 1 Faculty of Biology, Medicine and Health, The University of Manchester, Manchester M13 9PL, UK; 2 Manchester Academic Health Science Centre (MAHSC), Core Technology Facility, The University of Manchester, Manchester M13 9NT, UK

## Abstract

ATAC-seq is a recently developed method to identify the areas of open chromatin in a cell. These regions usually correspond to active regulatory elements and their location profile is unique to a given cell type. When done at single-cell resolution, ATAC-seq provides an insight into the cell-to-cell variability that emerges from otherwise identical DNA sequences by identifying the variability in the genomic location of open chromatin sites in each of the cells. This paper presents Scasat (single-cell ATAC-seq analysis tool), a complete pipeline to process scATAC-seq data with simple steps. Scasat treats the data as binary and applies statistical methods that are especially suitable for binary data. The pipeline is developed in a Jupyter notebook environment that holds the executable code along with the necessary description and results. It is robust, flexible, interactive and easy to extend. Within Scasat we developed a novel differential accessibility analysis method based on information gain to identify the peaks that are unique to a cell. The results from Scasat showed that open chromatin locations corresponding to potential regulatory elements can account for cellular heterogeneity and can identify regulatory regions that separates cells from a complex population.

## INTRODUCTION

Single-cell epigenomics studies the mechanisms that determine the state of each individual cell of a multicellular organism (1). The assay for transposase-accessible chromatin (ATAC-seq) can uncover the accessible regions of a genome by identifying open chromatin regions using a hyperactive prokaryotic Tn5-transposase (2,3). In order to be active in transcriptional regulation, regulatory elements within chromatin have to be accessible to DNA-binding proteins (4). Thus chromatin accessibility is generally associated with active regulatory elements that drive gene expression and hence ultimately dictates cellular identity. As the Tn5-transposase only binds to DNA that is relatively free from nucleosomes and other proteins, it can reveal these open locations of chromatin (2).

Epigenomics studies based on bulk cell populations have provided major achievements in making comprehensive maps of the epigenetic makeup of different cell and tissue types (5,6). However such approaches perform poorly with rare cell types and with tissues that are hard to separate yet consist of a mixed population (1). Also, as seemingly homogeneous populations of cells show marked variability in their epigenetic, transcription and phenotypic profiles, an average profile from a bulk population would mask this heterogeneity (7). Single-cell epigenomics has the potential to alleviate these limitations leading to a more refined analysis of the regulatory mechanisms found in multicellular eukaryotes (8).

Recently, the ATAC-seq protocol was modified to apply with single-cell resolution (3,9). Buenrostro *et al*. (3) used a microfluidic approach to isolate cells whereas Cusanovich *et al*. (9) avoided physical isolation of cells by using a combinatorial indexing strategy. However, neither of the studies developed a clear bioinformatics pipeline for the processing of the data and its downstream analysis. *chromVar* was the first Bioinformatics tool developed by *Schep et al*. to analyse scATAC-seq data (10). However, analysis in chromVar depends on the loss or gain of chromatin accessibility on a set of genomic features which could be either motif positions or genomic annotations rather than considering the full list of chromosomal locations. SCRAT (11) also uses a number of predefined features like Motif, Encode cluster, Gene and Gene sets to cluster cells into different sub-populations. This limits its application to chromosomal locations that represent an annotated feature. Furthermore, in scATAC-seq, chromosome accessibility is essentially a binary phenomenon due to the sparsity of data and uniqueness of a chromosomal location, and statistical approaches that are more appropriate for binary data should be used. Most of these tools do not have a processing pipeline and are not flexible enough to modify the functionality according to user requirements. The Octopus toolkit automates the mining of publicly available epigenomics and transcriptomics data in a single step (12) but lacks the functionality to process single-cell data. Linnorm is a robust statistical analysis method that has the advantage in speed and technical noise removal, but the method is suitable for single-cell RNA-seq only where the data is count data comprising of positive integer values and not binary values (13). A bioinformatics pipeline that has all the processing steps for scATAC-seq data, works directly on binary data and is flexible enough to easily incorporate user defined functionality is not yet available.

In this paper we introduce a bioinformatics pipeline called Scasat that can process sequencing data in a few simple steps while downstream analysis explicitly takes into consideration the binary nature of chromatin accessibility. This binarization approach is useful for identifying patterns of open chromatin in single cells but data with higher depth from bulk experiments are required to infer a network of upstream regulators. By incorporating a number of novel features, the Scasat pipeline is able to conduct the following tasks:

- Processing: Adapter trimming, quality control, mapping, removing blacklisted reads, removing PCR duplicates and calling peaks.
- Peak accessibility for each cell: We first merge all the single-cell BAM files to create a reference set of peaks. An accessibility matrix is then generated using this reference set with the accessibility information for these peaks in each of the cells.
- Quality Control: Lower quality cells and peaks are removed as well as peaks that may exacerbate batch effects.
- Downstream analysis: Clustering the cells to deconvolute cell types from a mixed cell population or identify differentially accessible peaks between two groups of cells.

**Figure 1.**
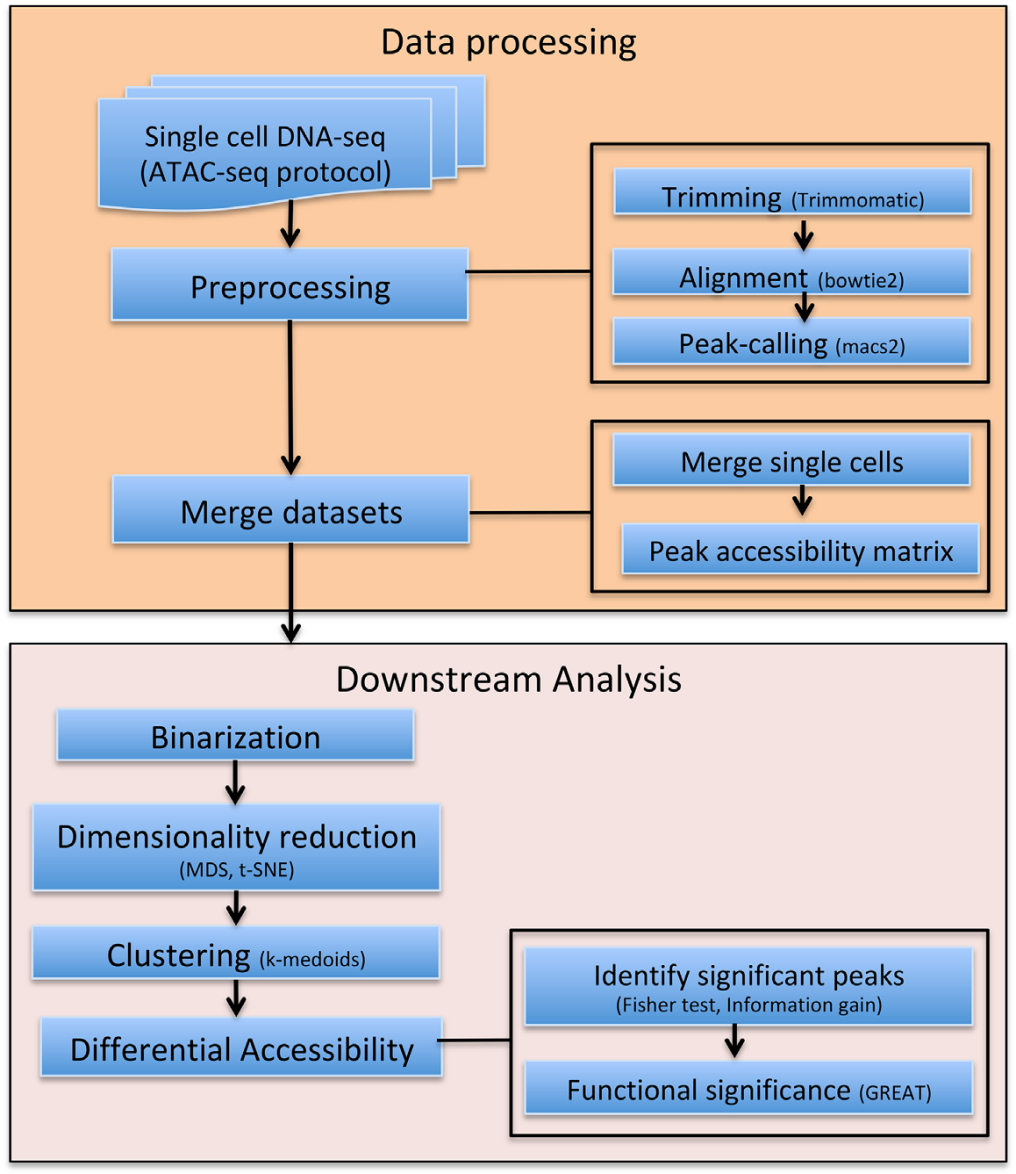
Complete work-flow of the Scasat. The data processing steps consist of the preprocessing steps of trimming low quality bases, aligning the reads with the corresponding genome and calling the peaks. The peak accessibility matrix is then generated by merging all the mapped BAM files of each single cell and calling peaks in this merged file. The downstream analysis is composed of the necessary steps to conduct statistical analysis on the single cell ATAC-seq data. In Scasat the data are converted into a binary dataset. Dimensionality reduction methods are then applied to this binarized data. Other statistical analysis is then performed on this reduced dimension to infer biological information.

Figure 1 describes the complete Scasat work-flow. In the following, we provide details of the workflow and demonstrate its utility by studying a mixture of three oesophageal tissue derived cell lines.

## MATERIALS AND METHODS

Here we describe, Scasat, **S**ingle-**c**ell **A**TAC-**s**eq **A**nalysis **T**ools, for processing and analysing single-cell ATAC-seq data. The Scasat workflow typically consists of four steps: (i) data processing; (ii) feature extraction; (iii) heterogeneity analysis of the cells; (iv) differential accessibility analysis of the peaks between two clusters of cells. To make it convenient for the user, we have introduced two notebooks for this analysis. The first notebook does the processing of the data and the second notebook does the downstream statistical analysis. Below we discuss the work-flow of both notebooks.

### Notebook environment

The complete pipeline was developed using the jupyter notebook environment (14). The jupyter notebook is a web-based notebook that can execute code, produce figures and put all the necessary explanations in the same place. As all the function definitions of the tool are open to the user, it can easily be extended to integrate new tools or approaches. Although the current version of the pipeline enables only the serial processing of the cells at the processing step, it can easily be extended for parallel processing. The notebook makes it easy to document, understand and share the code with non-technical users (15). Scasat uses many of the most widely used software tools at the processing step. The parameters and paths of these tools are set in the python environment. For the downstream analysis Scasat uses the R programming language for statistical computing and graphical visualization of the results. The use of R magic cells in the notebook variables makes the pipeline more robust and allows both programming languages to be used in the same workflow.

### Sequence data processing

The processing step starts with first configuring the folders and setting the paths of the software. The user configures the *inputFolder* to the foldername where all the *fastq* files are. The *outputFolder* is configured to store all the processed files. Experiments using sequencing applications (ATAC-seq, Chip-seq) generate artificial high signals in some genomic regions due to inherent properties of some elements. In this pipeline we removed these regions from our alignment files using a list of comprehensive empirical blacklisted regions identified by the ENCODE and modENCODE consortia (16). The location of the reference genome is set through the parameter *ref_genome*. This folder contains the index file for the *bowtie2* aligner. A brief description of the tools that we have used in this processing notebook are given below

- Trimmomatic v0.36 (17) is used to trim the illumina adapters as well as to remove the lower quality reads.
- Bowtie v2.2.3 (18) is used to map paired end reads. We used the parameter *–X 2000* to allow fragments of up to 2 kb to align. We set the parameter –dovetail to consider dovetail fragments as concordant. The user can modify these parameters depending on experimental design.
- Samtools (19) is used to filter out the bad quality mapping. Only reads with a mapping quality >q30 are only retained. Samtools is also used to sort, index and to generate the log of mapping quality.
- Bedtools intersect (20) is used to find the overlapping reads with the blacklisted regions and then remove these regions from the BAM file.
- Picard’s MarkDuplicate (21) is used to mark and remove the duplicates from the alignment.
- MACS2 (22) is used with the parameters –nomodel, –nolambda, –keep-dup all –call-summits to call the peaks associated with ATAC-seq. During the callpeak we set the *P*-value to 0.0001. This is due to the fact that otherwise MACS2 will not call the peaks having a single read mapped to it as it would consider those reads to be background noise.
- Bdg2bw is used to generate the Bigwig files for the UCSC genome browser visualization.
- QC: A final quality control is performed based on the library size of the BAM file. We filter out the cells for which the library size estimated by Picard tool is less than a user-defined threshold. The default value of the LIBRARY_SIZE_THRESHOLD is set to 10000. We consider any cell having a library size lower than this threshold to not be a valid cell as those reads may come from debris free material or from dead cells.

### Downstream analysis

Single-cell ATAC-seq is essentially binary in nature. A specific location in a chromosome for a specific cell can either be open or closed. In contrast to bulk data where more reads aligning to a specific location of a chromosome would indicate more cells in the population having open chromatin at that location, in single cells it could only be due to the multiple insertions in that region or possibly other alleles at that locus. As described by Buenrostro *et al*. (3) such reads are rare for single-cell ATAC-seq which is overwhelmly dominated by single reads for a specific location of a chromosome for each individual cell. Binarising the data might have a minor impact only on the small proportion of chromosomal locations with multiple reads. As it does not remove any peak, this should not make an impact on detecting sub populations.

We merge the single-cells to get the list of reference peaks. Scasat then binarizes this peak information for each individual cell for downstream analysis. One of the strengths of Scasat is that the statistical approaches used in the analysis pipeline take into consideration the binary nature of chromatin accessibility in single cells. For batch correction we did not use conventional methods like Combat (23) or *removeBatcheffect* from Limma (24) as the tools convert the batch corrected data into real values. Instead we devised our own batch correction method that keeps the data binary while correcting for batch effects.

### Peak accessibility matrix

The analysis workflow of Scasat starts by merging all the single-cell BAM files and creating a single aggregated BAM file. Peaks are called using MACS2 on this aggregated BAM file and sorted based on *q*-value. Peaks in this list are the ones that are open in at least one single-cell. Using this list of peaks we generate the *peak accessibility matrix*. The rows of this matrix represent all the peaks from the reference set and the columns represent each single cell. The pipeline calculates the accessibility of the peaks for each individual cell where it has at least one overlapping read and encodes it as a binary value. Creating this pre selected list of ensemble peaks is necessary for having an aggregated peak list that shows the overall behaviour of the dataset. While calling peaks in the aggregated BAM file might get rid of some peaks with much lower statistical significance (open only in very few cells), again this can be circumvented by sampling more cells.

For each individual cell, peaks that overlap with this list of accessible regions are given the value of 1 in the table. For all the other peaks it is 0. A graphical explanation of this process is given in Supplementary Figure S1.

### Bulk versus aggregate

If a bulk measurement is available for the same cell-type or sample, then the pipeline can calculate *Number_of_peaks* versus *precision* for the aggregated single-cell data against its population-based bulk data. This demonstrates how closely the single-cell data recapitulates its bulk counterpart. We define *peakA’s* list as all the peaks in the population based on bulk data and *peakB’s* list as the peaks in aggregated single-cells sorted on *q*-values. *peakA* is considered to be the gold standard for this calculation. We start with the top 100 peaks in the sorted peak list of *peakB*. All the peaks within this 100 list that overlap with the peaks in *peakA* are considered True Positive and the ones that do not overlap are False Positives. We then calculate the precision as

(1) \begin{equation*} {\rm Precision} = 100 \times \frac{{\rm TP}}{{\rm TP} + {\rm FP}} \end{equation*}

where TP = True Positive, FP = False Positive, TN = True Negative. We then repeat the process by increasing *peakB* by 50 peaks each time while keeping *peakA* fixed. This gives us a *Recall* versus *Precision* plot show in Supplementary Figure S2.

### Filtering Peaks

Peaks appearing in a small number of cells are less informative and are not always appropriate for downstream analysis. Similarly, some cells passing through library size filtering, might still have a very low number of peaks. In our downstream analysis we filter out these cells and peaks. If a cell has open peaks below a user defined threshold (default: 50 peaks) in the *peak accessibility matrix* we would remove that cell from subsequent analysis. Also peaks not observed across a user defined number of valid cells (default: 10 cells) are not considered for further downstream analysis. Choice of this threshold depends on the experimental design, the nature of the cells and other biological as well as technical factors. Users need to carefully define these thresholds for filtering based on their experimental design.

### Calculate Jaccard distance

Once the accessibility matrix is generated, we are interested in a dissimilarity measure that quantifies the degree to which two cells vary in their peak accessibility. In our pipeline we use the Jaccard distance (25) as a dissimilarity measure. The Jaccard distance is the ratio between the number of peaks that are unique to a cell against all the peaks that are open in two cells.

### Dimensionality reduction

Our *peak accessibility matrix* represents a very high-dimensional dataset of open chromatin regions for each single-cell. Dimensionality reduction for this high-dimensional dataset is essential for easy visualization and other downstream analysis. In our pipeline, we applied multidimensional scaling (MDS) and t-distributed stochastic neighbour embedding (t-SNE). MDS provides a visual representation of similarity (or dissimilarity) between two objects. It takes as input the distances between any pair of objects and then minimizes a loss function called strain (26) so that the between object distances are preserved as much as possible. t-SNE is a non-linear dimensionality reduction technique that maps multidimensional data to two or more dimensions for easy visualization. t-SNE converts the similarity between the data points to joint probabilities and tries to minimize the Kullback–Leibler divergence between the joint probabilities of high dimensional data and low-dimensional embedding of this data (27). To reduce the noise and computational time it is recommended to use a lower dimensional representation of the data as input to t-SNE, e.g. from MDS. It is worth noting that the number of dimensions that are taken as input for t-SNE have an effect on the way the clusters are visualized on the t-SNE plot. Depending on the complexity of the samples users can choose between 10 and 50 dimensions as t-SNE input.

### Clustering

In this pipeline, we used the *k*-medoids algorithm to cluster the cells into different groups. The *k*-medoids algorithm breaks the dataset into different partitions and attempts to minimize the distance between points assigned in a cluster and a point designated as the center of that cluster. In our pipeline we used Jaccard distance as the dissimilarity matrix for this algorithm.

### Differential accessibility (DA) analysis

One of the key features of interest in single-cell ATAC-seq analysis is to perform a statistical analysis to discover whether quantitative changes in accessibility of chromatin locations between two groups of cells are statistically significant. The pipeline implements two methods, Fisher exact test and Information gain, to conduct the differential accessibility analysis between any two groups of cells.

#### Information gain

Based on the expected reduction in entropy (homogeneity measure), information gain measures the attribute that provides the best prediction of the target attribute where entropy is reduced. In differential accessibility analysis, *information gain* lists the peaks that best splits the cells into different groups. Defining entropy as $\sum _{i=1}^c -p_i \log_2\, p_i$, the Information gain is calculated as

\begin{equation*} {\rm Gain}(P, P_{G_1}, P_{G_2}) = {\rm Entropy}(P) - \sum _{v \epsilon \lbrace G_1,G_2 \rbrace } \frac{|P_v|}{|P|}{\rm Entropy}(P_v) \end{equation*}

Here, *P* is the collection of all data, $P_{G_1}$ represents the cells in *G*_1_ and $P_{G_2}$ represents cells in *G*_2_. We calculate the information gain of each of the peaks given the cells are divided into two groups. The peaks are then sorted based on the information gain and the user can choose the cutoff value for selecting the DA peaks.

#### Fisher exact test

The Fisher exact test looks at a 2 × 2 contingency table that shows how different groups/conditions have produced different outcomes. Its *null hypothesis* states that the outcome is not affected by groups or conditions. We run this test on a peak-by-peak basis by organizing the open and closed (1’s and 0’s) for each peak in a 2 × 2 contingency table. The p-values are then corrected using Bonferroni correction for multiple comparisons (28). Differentially accessible peaks with statistical significance are then selected based on a user-defined cutoff value (default: *q*-value <0.01).

**Figure 2.**
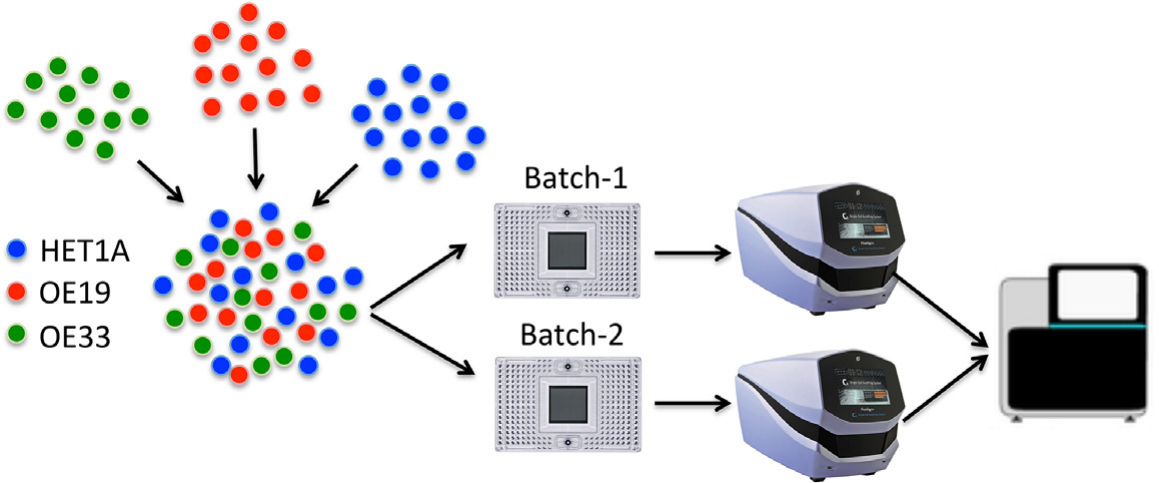
Experimental design for disentangling different cell types from a complex mixture. Three cell types HET1A, OE19 and OE33 are mixed in equal proportions. Samples are then taken from this mixture into two independent batches (Batch-1 and Batch-2) by running them in two C1 integrated fluidic circuits (IFC). The captured cells are then sequenced using a NextSeq.

### Experimental design

To demonstrate Scasat we generated scATAC-seq data from a mixture of three different cell types and the objective was to identify these three cell types from this mixture. We applied Scasat to characterize biologically relevant chromatin variability associated with each cell-type. To create the mixed cell population we took two classic oesophageal adenocarcinoma (OAC) cell lines, OE19, OE33 and one non-neoplastic HET1A cell line. We mixed the three cell types in equal proportions to create a heterogeneous population (see Figure 2). Two samples from this mixture were taken to make two technical replicates. ATAC-seq was then performed on those two replicates by loading on two separate C1 fluidigm chips using a 96-well plate integrated fluidic circuit (IFC) and sequenced on an Illumina NextSeq ( Figure ). As ATAC-seq reports on the accessible regions of the chromatin which are considered to be active (29), these three cell lines are expected to have different accessibility at regulatory regions. The analysis attempted to disentangle these cells based on the presence of these active sites.

We benchmarked Scasat with single cell ATAC-seq from Buenrostro *et al*. (accession number: GSE65360) (3). There are in total 1632 samples with 1920 runs. For samples that have multiple runs we kept the runs with more sequencing data. We then filtered out the mouse data and only took the human cells. These cells were then pre-processed with our Scasat pipeline and filtered based on Library size. After this filtering, we have 743 cells for downstream analysis.

We also benchmarked Scasat with single cell ATAC-seq from Cusanovich *et al*. (30). We randomly sampled 3000 cells from this single cell atlas and applied Scasat to visualize the cells in a t-SNE plot.

### Sample preparation

We plated 1 × 10^6^ of each cell type onto 10 cm plates and incubated for 24 h at 37°C. Cells were detached using 0.1% trypsin. After detaching cells, each individual cell type was placed into a separate tube, centrifuged and re suspended in 1 ml of 1× phosphate-buffered saline (PBS). Each cell type suspension was quantified using a haemocytometer and this gives us a concentration in cells per *ml*. We then put the same number of each cell type into the same tube (to get the mixed population), which was then centrifuged again and re-suspended in 100 μl of 1× PBS. This was then submitted to the Genomic Technologies Core Facility. The C1 platform (Fluidigm) was used to capture single cells and generate sequencing libraries using the scATAC-seq protocol available from the Fluidigm ScriptHub. Medium (10-17 micron) C1 for OpenApp Integrated Fluidic Circuits (IFCs) were used to capture and process the samples. The amplified DNA products were harvested, and then additional PCR performed to dual-index the harvested libraries using customized Nextera PCR primer barcodes (IDT Technologies) according to the ScriptHub protocol. The PCR products were then pooled to a total volume of 96 μl, followed by two cycles of AMPure XP bead purification (Beckman Coulter, Inc.) according to the SMART-seq v4 protocol 032416 (Clontech Laboratories, Inc.) using C1 Dilution Reagent (Fluidigm) for the final elution. The pools were then quantified and validated using qPCR-based KAPA library quantification kit for Illumina (KAPA Biosystems) and TapeStation 4200 (Agilent Technologies). The library preps are done as part of cell capture on C1 plates, so for both the batches they are prepared separately. Library pools (1.8 pM final concentration) were then sequenced (75:75 bp, paired-end) on the NextSeq500 platform using the Mid Output v2 150 cycle kit (Illumina, Inc.) to generate .fastq sequence files. The C1-runs were done with different batches of cells and across different days, so they are biological replicates. Both batches were made with the same proportion of cells. Both the batches are then sequenced on the same Nextseq run. We were surprised by the batch effect but it also shows the power of our pipeline to overcome these big effects and successfully separate the cell types.

## RESULTS AND DISCUSSION

First we processed the sequencing data to generate a peak accessibility for each single cell where a binary score is given to each potential peak in the population. The two batches were processed separately making it easier to keep track of the processing steps and to troubleshoot any problem that might arise due to batch effects. The parameters for the tools are explained in the tool description section. We trim the adapter sequences using Trimmomatic and used Bowtie2 to map reads to the genome. After removing the Blacklisted regions, we used *Picard’s Markduplicate*, to remove the duplicates. We then removed the chrY (as the three cells lines are a mixture of male and female) and chrM. The peak calling was done in two stages. In the first stage *macs2* was used to call peaks in each individual cell by setting the *P*-value parameter to 0.0001 to ensure that peaks associated with low mapping reads are also called. We then filtered the cells that fail to cross the LIBRARY_SIZE_THRESHOLD set to 10 000. After this QC, Batch-1 has 84 and Batch-2 had 89 cells for downstream analysis. In the second stage of peak calling, we aggregated all the BAM (both batches) files by calling *getAggregatedPeak()* module and used *macs2* to call peaks on this aggregated BAM file with *q*-value of 0.2. This gave us a reference set of peaks. We then used the *mergePeaks()* module to merge the overlapping peaks in this reference set and sort them based on the *q*-value giving us a total of 236 580 peaks as reference set. Finally we used the *peakAccessibility()* module to calculate the accessibility of these reference peaks for each single-cell and generated the *peak accessibility matrix*.

### Peak selection

Next we identified and removed peaks from these matrices which likely represent background noise. We used the clean.count.peaks(min.cell.peaks.obs=10, min.peaks.cell=50) with the default parameters to remove the lower quality cells and peaks.

In Scasat, the threshold for filtering the cells and peaks depends on the experimental design ie. cell types, sequencing depth or batches if there are any. For this reason the users can tune these filtering parameters to an appropriate value suitable for their experiment. In general, with smaller number of cells (within 200 cells) we set the threshold on *min.peaks.cell* to 50 peaks, that is a cell has to have at least 50 opening peaks to call it a valid cell and *min.cell.peaks.obs* to 10 meaning a peak has to be observed in at least 10 cells to be a valid peak. When the number of cells is higher, it is suggested to set *min.peaks.cell* to 0.1% of total accessible peaks and for valid peaks they should be present in at least 2% of the cells (min.cell.peaks.obs).

These peaks are filtered out because we believe they do not contain much information for reliable statistical inference. One caveat of this approach could be that peaks associated with more abundant cell types are potentially kept and we might fail to detect rare sub-populations. However, we think that in the majority of cases, even if some of these peaks represent real biological signals and represent a rare sub-population, removing them would still not be a problem as the open chromatin locations operate on a complex network of interactions and other more informative peaks would be able to pick up these rare cell-types if they exist. A pragmatic approach to completely circumvent this problem of missing rare sub-population would be to sample a larger number of cells. The recent evolution of single cell ATAC-seq protocols to high throughput commercially available platforms makes this a feasible solution. As these peak selection thresholds are now adjustable, the user can alter them based on their experimental design and number of cells.

**Figure 3.**
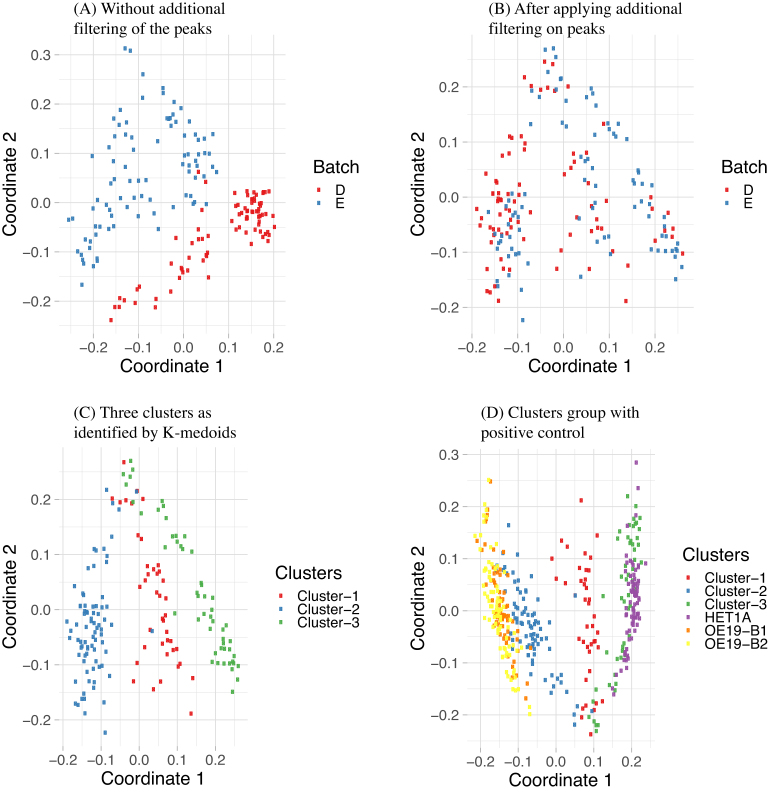
MDS plot with (**A**) and without (**B**) additional filtering to remove peaks where very few cells from a specific batch have open peaks. In (A) cells can be separated using only the batch information which is removed in (B) with additional filtering. (**C**) shows the three clusters identified by *k*-medoids clustering algorithm. (**D**) MDS plot derived from the mixed cell dataset using cells from clusters 1–3 (C) and cells from experiments conducted with pure populations of HET1A and OE19. B1 and B2 in OE19 refers to two batches of OE19 single cells. The clusters from the mixed population group with their inferred cell-types.

We applied MDS on these filtered peaks to reduce the dimensionality of the data and plotted them in a 2D plot (Figure 3A). In Figure 3A, we saw that the cells are separated based on their batches indicating a clear batch effect. We cannot apply conventional batch correction methods as that gives normalized data that violates our assumption of binary data. Therefore, in order to remove the batch effect we applied an additional filtering. Careful analysis of the data found that the peaks in Batch-1 had higher number of zeros associated with aggregated peaks, indicating that less information is attained from Batch-1 cells compared to Batch-2 (Supplementary Figure S2). We therefore applied an additional filtering. In this filtering, we removed the peaks that are open only in one batch meaning that for those peaks no information is coming from the other batch. If a peak is open in less than three cells in a single batch then we removed this peak. This further reduced the number of peaks to 37,071 peaks. After this filtering we again applied MDS and plotted it in a 2D plot. Now we can see that the cells are no longer separated based on their batches (Figure 3B). We next applied *k*-medoid clustering on the remaining peaks and find three nicely separated clusters (Figure 3C). We then combined this data with three single cell datasets where the cell-types are known, one HET1A and two OE19 cells from two batches, batch B1 and B2. Additional filtering is applied in this combined dataset to remove the peaks that are only open in less than three cells in any specific single cell-type. We then applied MDS with this combined data and results are shown in Figure 3D.

### Clustering analysis of the cell populations

To attempt to partition the cell mixture into individual cell types, we clustered the peak accessibility data. We used the *Partitioning Around Medoids (PAM)* algorithm, the most common realisation of k-medoid clustering to cluster the cells. For this algorithm we chose *K* = 3 as we expected the cell-mixture to have three types of cells. While calculating the partitions we passed the Jaccard distance to the function. The assignment of the clusters for each of the cells are superimposed onto our MDS plot which is shown in Figure 3C. Cluster 1 has 41 cells, Cluster 2 has 80 cells and Cluster 3 has 52 cells.

### Identifying cell types

In Figure 3D, we see that the cluster-2 cells group with OE19 cells and Cluster-3 groups with HET1A cells. Cluster-1 remains in the middle without grouping with any cell-types. From this we infer that Cluster-1 is composed of OE33 cells, Cluster-2 contains OE19 cell and Cluster-3 is likely composed of HET1A cells.

To further validate our inference of the different clusters, we compared them with the bulk data of HET1A, OE19 and OE33 and also with two single-cell datasets from the same cell types.

**Figure 4.**
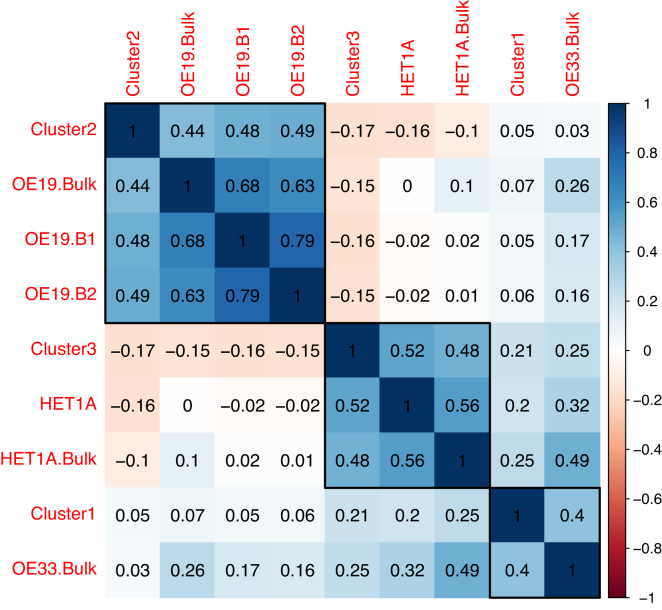
Correlation plot showing Pearson correlation for the three clusters of cells identified with k-medoid clustering algorithm, against the bulk and two other single-cell data for which the cell types were known *a priori*. These known single-cell data are for HET1A and OE19 done in two batches (B1 and B2). Single cells from these known cell types are also aggregated. The bulk datasets have the label bulk at the end of the sample name. Here red indicates negative correlation and blue indicates positive correlation (scale bar on the right).

For this comparison, we merged the mapped reads for each cell in each of the clusters to create three aggregated BAM files, one for each of Cluster-1, Cluster-2 and Cluster-3 identified in Figure 3C. For the other two single-cell datasets from a different experiment we did the same. We now extend the column of *peak accessibility matrix* by calculating the peak accessibility for the three aggregated single cell data (OE19 B1, B2 and HET1A) and the bulk datasets (HET1A, OE33, OE19) using the same 37,071 peaks which are left after removing the low quality peaks and peaks only associated with a specific batch. Finally, we calculated the Pearson correlation coefficient for each of these datasets against each other which is shown in a correlation plot (Figure 4) where the cell-types are clustered based on hierarchical clustering. The correlation plot assigns each of the single cell clusters to their respective cell type based on high correlation coefficient with the known cell types. In the correlation plot (Figure 4), Cluster-2 groups with OE19 cells. It has a correlation coefficient of 0.48 and 0.49 with the OE19 single-cells (OE19.B1 and OE19.B2) and 0.44 with the OE19 Bulk dataset (OE19.Bulk), identifying Cluster-2 as OE19 cell-type. Cluster-3 groups with HET1A cells, where it has a correlation of 0.52 with the HET1A single cell and 0.48 with HET1A Bulk dataset (HET1A.Bulk), identifying Cluster-3 as HET1A cell-type. Finally we infer Cluster-1 to be of OE33 cell-type as it has correlation coefficient of 0.4 with OE33 Bulk data. If the bulk data is not available or any other input cell types are not known, GO based analysis of genes associated with open chromatin peaks, e.g. GREAT (31) can be used to aid the assignment of cells to a particular cell type.

### UCSC genome browser visualization

**Figure 5.**
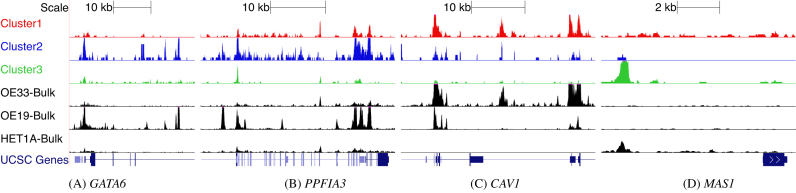
Confirmation of our assignments through visualizing peaks across different genome location from UCSC genome browser track. The clusters in three different colors (red, blue, green single cell clusters) correspond to the inferred aggregated single-cell peak pattern. The bottom three tracks in black show the peaks for the bulk data of OE33, OE19 and HET1A respectively. Peaks around the gene body of (**A**) GATA6, (**B**) PPFIA3, (**C**) CAV1, (**D**) MAS1 are shown in the figure. For these genes, peaks are more open in (A) and (B) OE19 cells, (C) OE33 and (D) HET1A cells.

To further validate the deconvolution of our mixed population into known cell types we visualize the ATAC-seq data of the clusters in the UCSC genome browser. We created a genome browser session of the aggregated single cells in the clusters and compared this to the data from bulk ATAC-seq experiments performed on known cell populations. The activation of *GATA6* can sustain oncogenic lineage survival in esophageal adenocarcinoma (32). This is consistent with the regulatory elements for this gene to be more open in our Cluster-2 cells and the OAC-derived OE19 cell line bulk data. In Figure 5(A) we see that *GATA6* are more open in Cluster-2 and OE19 bulk data. This further indicates that Cluster-2 is comprised of OE19 cells. Another gene that is expressed in OE19 cells is *PPFIA3* (33). The peaks associated with *PPFIA3* gene in Figure 5(B) are open in Cluster-2 and have a very similar opening pattern to OE19 cells. *PPFIA3*, is a gene encoding a receptor that has been reported to show moderate cytoplasmic activity in colorectal cancers (34) which can develop into oesophageal metastasis (35). We also ran our differential accessible analysis on Cluster-1 vs Cluster-2 peaks and found that the regulatory region of *CAV1* is differentially accessible between Cluster-1 and Cluster-2. *CAV1* is a tumour suppressor gene candidate (36) and has higher chromatin opening in OE33 cells. We see the same behaviour of *CAV1* for both Cluster 1 and OE33 bulk data in our genome browser (Figure 5C). This confirms Cluster 1 as OE33 cells. Britton *et al*. (37) reported an intragenic open chromatin location at the *MAS1* locus for HET1A (37) which we also observe in Figure 5D for both our Cluster-3 and HET1A cells, confirming the identify of Cluster-3 cells as HET1A cells.

### GO based functionality

**Figure 6.**
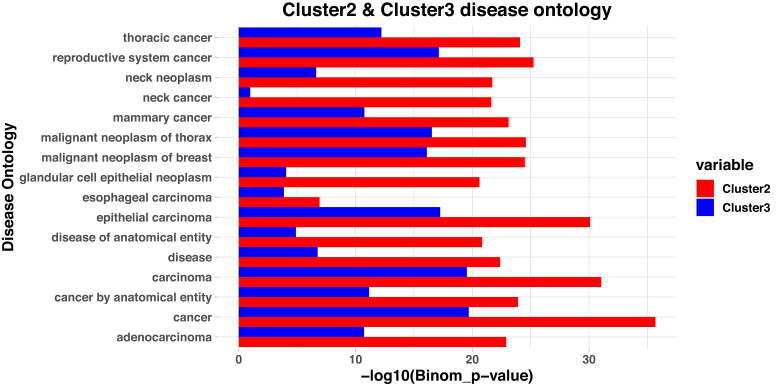
Disease ontology from the differentially accessible peaks in Cluster-2 compared to Cluster-3. We identified all the peaks that are differentially accessible in Cluster-2 with a log_2_FoldChange of more than two. We then identified the disease ontologies that are associated with these peaks. Similarly we identified the peaks that are differentially accessible in Cluster-2 compared to Cluster-3 with positive log_2_FoldChange (more open in Cluster-2) and identified the disease ontologies associated with these peaks. We then took the 15 topmost disease terms associated Cluster-2 based on p-values along with esophageal carcinoma as two cell lines are related with esophageal carcinoma. The Binomial p-values for these peaks in both clusters are plotted in the –log_10_ scale here. All the diseases that have very high confidence in Cluster-2 are related with cancer. For these same disease, there is much lower statistical significance in Cluster-3. For esophageal carcinoma, the statistical significance is higher in Cluster-2 compared with Cluster-3 further supporting the conclusion that Cluster-2 is mostly composed of OE19 cells and Cluster-3 is mostly composed of HET1A cells.

To confirm these cluster assignments further, we looked at the disease ontology associated with the cells in the clusters. We ran our differential accessibility analysis with the module *getDiffAccessInformationGain* which uses the entropy and information gain to identify the differentially accessible peaks between *Cluster-2 vs Cluster-3*. We used the peaks that we identified during differential accessibility analysis of Cluster-2 and Cluster-3 and took the peaks that are differentially accessible with positive log_2_ fold change (more open) in Cluster-2 compared to Cluster-3 and identified the Disease ontologies associated with these peaks through GREAT. GREAT first associates the peaks with potential target genes by finding the nearest gene to TSS and then identifies the disease ontologies associated with these open chromatin locations. The same process was repeated for Cluster-3 where we identified the disease ontologies associated with open chromatin locations of Cluster-3. We then took the 15 topmost disease-related terms associated with Cluster-2 and determined the statistical significance for these terms in the open peaks found in Cluster-3. In addition we considered esophageal carcinoma as two cell lines are related with this. The −log_10_(Binomial_p-value) for these diseases are shown in Figure 6. Esophageal carcinoma is picked up as one of the diseases associated with Cluster-2 with higher confidence compared to Cluster-3 cells. Several other significant disease associations are seen with adenocarcinomas and with general cancer. In all of these disease ontologies Cluster-2 shows high statistical significance compared to Cluster-3. This further confirms our accurate identification Cluster-2 as a cancer derived subtype whereas Cluster-3 lacks these features as would be expected for the non-tumorigenic HET1A cells.

### Benchmarking with other datasets

Next we asked whether our method could be successfully applied to other scATC-seq datasets generated by different different groups using a variety of different methodologies. Scasat successfully separated different cell-types in the Buenrostro dataset as shown in the t-SNE plot in Supplementary Figure S4A and B. To cluster the cells in this dataset we applied Dynamic Cut Tree which applies a novel dynamic branch cutting method to detect clusters in a dendrogram (38). A major advantage of this method is that we do not need to select the number of clusters beforehand. The clustering identifies a slightly higher number of clusters than the number of known cell types from Buenrostro *et al*. (3). These clusters can then be merged manually based on expert knowledge while looking at the binding motifs associated with the clusters. We also applied Scasat to characterize the K562 cell type but first identified the differentially accessible peaks that are more open in K562 cells compared to all other cells and then associating disease ontology terms with these peaks. We know that K562 cells are a human myelogenous leukemia cell lines and this is the disease ontology picked up from the first 2 disease ontology in the GREAT analysis (Supplementary Figure S5) confirming our pipeline’s effectiveness in characterizing the cells correctly. Next we analysed the scATAC-seq data generated from 13 adult mouse tissues (30). Supplementary Figure S6 shows the visualization of 3000 randomly selected cells in a t-SNE plotcoloured according to tissue of origin. At first we generated a t-SNE plot taking 10 MDS dimensions as input. Although cells from major tissue types were nicely separated in this plot, some closely related cells like testis and small intestine stayed together. However, when we increased the number of MDS dimensions to 40 as input to t-SNE, this nicely separated all the different tissue types including the testes and small intestine. The t-SNE plot has a blob in the middle with a mixture of different cell types which is also observed in the t-SNE plot of Cusanovich *et al*. Like Cusanovich *et al*. we also observe four different clusters of brain tissues which are again mixed with cells from whole brain and cerebellum. However one shortcoming with our approach is that we tested our method on a much smaller number of cells compared to Cusanovich *et al*. This causes the method to have less predictive power for clear cell separation, for example in the case of cells from lung tissues we do not see a very clear cluster of cells.

Together, these results illustrate the utility of our methodology for analyzing a wide range of datasets generated using different platforms and experimental protocols.

## CONCLUSION

As single-cell ATAC-seq experiments are gaining momentum, we report Scasat, a pipeline to process and analyse single-cell ATAC-seq. Scasat offers two major utilities, the initial processing of scATAC-seq data and its downstream analysis. Scasat is implemented in jupyter notebooks making it simple, robust, scalable and easy to extend. The tool also incorporates novel analytical capabilities of addressing the open chromatin information as binary data, and correcting for the batch effects while keeping the binary nature of the data. This gives Scasat an advantage over other tools in analyzing single cell ATAC-seq data. Also, we addressed the differential accessibility problem from a totally different perspective where we used entropy and information gain to calculate the differentially accessible peaks between two sets of cells. We applied Scasat to a mixture of cells to deconvolute those cells into different groups. Initial results from our analysis showed that batch effects confounded the biological signal which hindered the classification of cells into different cell types. Therefore Scasat was further developed to overcome this batch effect problem. Final results showed that an unsupervised clustering of the cells based on accessible chromatin regions could group cells into their corresponding cell type. This suggests that regulatory elements can define cell identity quite precisely. Similar approaches could be applied to separate malignant cells from normal cell in a cancerous tissue and then investigate the malignant cells in detail. Scasat was also successfully applied to publicly available datasets showing its overall utility for processing and analyzing single-cell ATAC-seq datasets generated using different protocols and platforms. The successful implementation of this tool helps us to further understand the epigenetic mechanisms at the single-cell level and opens opportunities for easier and better analysis of single-cell ATAC-seq data.

## SOFTWARE AND DATA AVAILABILITY

Jupyter notebooks with the complete pipeline applied to the dataset published with this paper along with another published dataset are publicly available on Github (https://github.com/ManchesterBioinference/Scasat). The fastq files are submitted at ArrayExpress (accession number E-MTAB-6116). The UCSC genome browser track can be accessed through https://goo.gl/BxJ95G

## Supplementary Material

Supplementary Data
