# Supplementary material for: Classifying cells with Scasat, a single-cell ATAC-seq analysis tool
Source: Nucleic Acids Res. 2018 Oct 18;47(2):e10. doi: 10.1093/nar/gky950 (PMC6344856; doi:10.1093/nar/gky950)
Supplement: Supplementary Data [file gky950_supplemental_files.pdf]

Supplementary Material for
Classifying cells with ScAsAT - a tool to analyse single-cell ATAC-seq

Syed Murtuza Baker[1], ∗, Connor Rogerson[1], Andrew Hayes[1], Andrew D. Sharrocks[1,2] and Magnus Rattray[1,2,∗]
[1] *Faculty of Biology, Medicine and Health, The University of Manchester, Manchester, M13 9PL, United Kingdom.*
[2] *Manchester Academic Health Science Centre (MAHSC), Core Technology Facility, The University of Manchester,Manchester, M13 9NT, United Kingdom.*

(Dated: September 25, 2018)

Abstract

This is supplementary material for Classifying cells with ScAsAT - a tool to analyse single-cell ATAC-seq. ScAsAt processes and analyzes the single-cell ATAC-seq data.

Recall vs Precision

Recall identifies the proportion of positives that a model identifies correctly whereas precision identifies the proportion of positive responses that are actually positive.

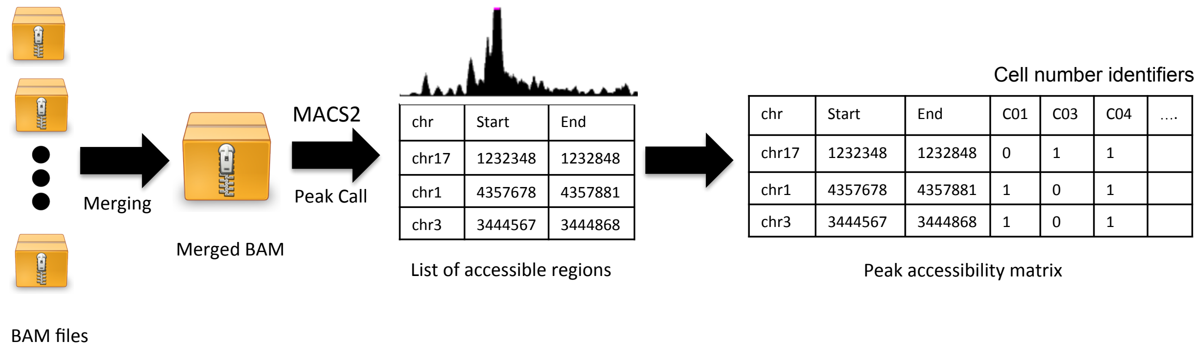

FIG. S1 Steps to generate peak accessibility matrix: BAM files of each of the cells are merged to generate the merged BAM file. MACS2 is used on this merged BAM file to generate the list of peaks that are shared across the single-cells. Peaks that overlap in this peak list for a cell are set the value of 1. All the other values are set to 0

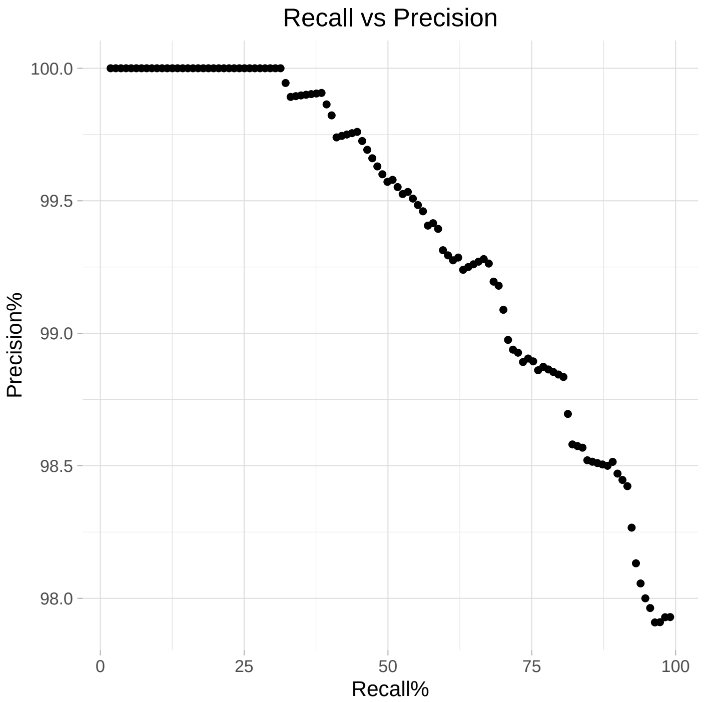

FIG. S2 Recall vs precision for cluster2 with OE19 bulk cells. Recall vs precision captures the relevance of cluster2 peaks with OE19 peaks. Precision which is also called positive predictive value, identifies the fraction of peaks in cluster2 that are actually OE19 peaks ie. it identifies the portion of True Positives in cluster2. Recall, also known as sensitivity, identifies the fraction of peaks in cluster2 that have been correctly identified as OE19 among all the peaks present in the bulk OE19 peaks. We start with 100 peaks in cluster2 with an step size of 50 peaks as well. Both the precision and peak for cluster2 against OE19 is very high indicating successful identification of our clusters with their corresponding cell types.

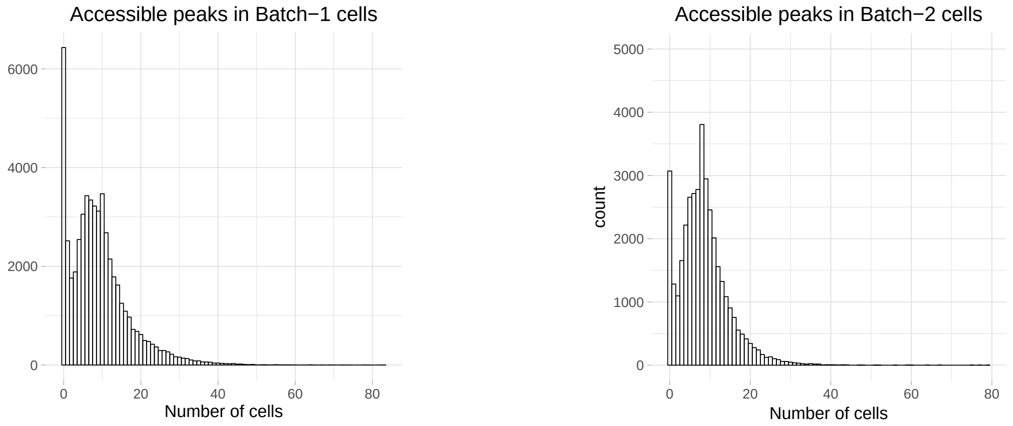

(A) Distribution of accessible peaks in cells in Batch-D (B) Distribution of accessible peaks in cells in Batch-E

FIG. S3 Histogram showing the distribution of peak counts for batch-1 and batch-2 in 26,089 peaks that we got after the initial filtering. Ones are counted for each of the peaks in all the cells in a specific batch to see in how many cells a specific peak is open. The x-axis shows the number of cells a peak is observed and y-axis shows the counts of the peaks. For eg. in Batch-1 4857 peaks are not observed in any of the cells and so we see a bar at 0 cell. Then 1262 peaks are open in exactly one cell which is visualised with the next bar on top of number of cells equal to 1.

Time and Resources required for pre-processing

We ran the mixed population data of 192 cells in a desktop machine with Intel Xeon CPU @ 3.00 GHz with 4 cpu cores and 64GB memory. All the processing for this data were done serially, meaning all the steps of the pre-processing for a cell is finished before starting the next one. It took 303 minutes for all 192 cells pre-processing to complete.

For Buenrostro data, we selected 1400 human cells for benchmarking Scasat. We ran the pre-processing of this dataset in a cluster node with Intel Xeon CPU E5-2640 v3 @ 2.60 GHz with 32-core. We ran the pre-processing with 4 parallel runs where each software was allowed to run 4 threads within their run.

Batch information

The C1-runs were done with different batches of cells and across different days, so they are biological replicates. Both batches were made with the same proportion of cells. The library preps are done as part of cell capture on C1 plates, so for both the batches they are prepared separately.

Cell capture information

The following table gives cell capture information based on visual annotation. However, as the images were not conclusive in majority of the time we did not use this table to filter out the cells rather used library size for filtering out the cells. Both the batches are then sequenced on the same nextseq run.

We were surprised by the batch effect but it also shows the power of our pipeline to overcome these big effects and successfully separate the cell types.

Batches	Single cells	Doublets	Multiplets	Empty/Not certain	Peak Statistics
D	80	7	3	6	
E	85	5	1	5	

Benchmarking Scasat

Scasat is applied to benchmark two single-cell ATAC-seq datasets. One is the Buenrostro et al 2015; doi:10.1038/nature14590 in which we considered only the human cells. The other is the Cusanovich et. al. 2018; doi:10.1016/j.cell.2018.06.052. We randomly selected 3000 cells from this cell atlas and applied Scasat to visualize the cell separation.

Benchmarking with Buenrostro dataset

We benchmarked human cells singe-cell ATAC-seq from Buenrostro et al 2015 and recapitulate the cell-types that Buenrostro identified. We then characterized some of the cell-types with the differentially accessible peaks identified with our differential accessible peak identification method.

For this benchmarking we took the human cells only. After filtering based on library size and peak information, there were 740 cells left for downstream analysis. We applied Scasat to visualize the cells in a t-SNE plot Figure ??(A & B). We also clustered the cells using an unsupervised clustering algorithm Figure ??(C). We then applied Scasat to characterize one K562 cell type Figure ??.

Benchmarking with Cusanovich dataset

We benchmarked Scasat with the dataset from Cusanovich et. al. 2018 where they build a single cell atlas for mammalian cells. We randomly sampled 3000 cells from this cell atlas and applied Scasat to visualize the cells in a t-SNE plot.

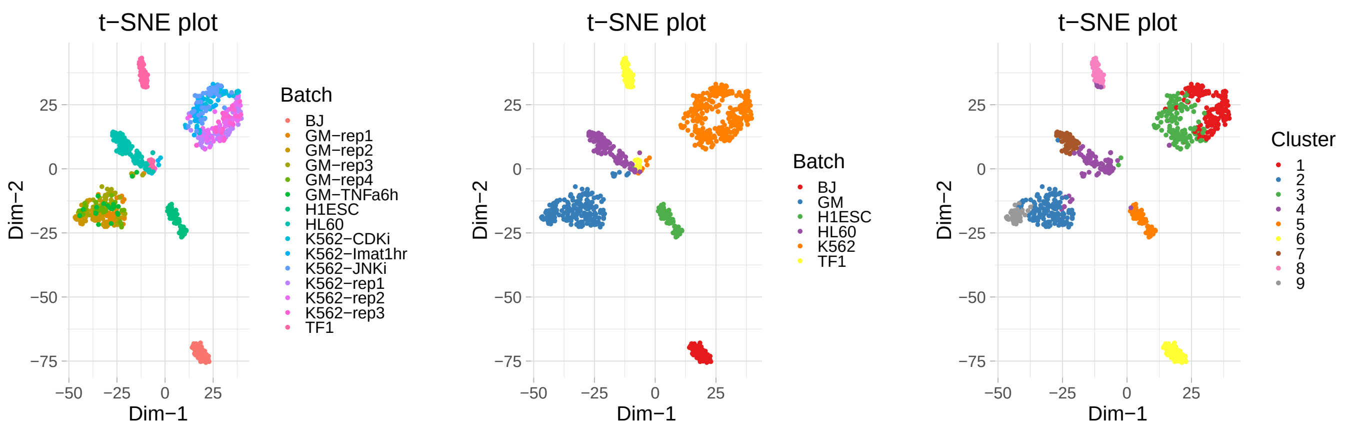

(A) t-SNE plot of individual replicates (B) t-SNE plot with replicates merged. (C) t-SNE plot with clusters

FIG. S4 t-SNE plot with 740 cells from Buenrostro et. al. dataset. (A) plots the cells with each individual replicates. Scasat nicely groups the cells in the t-SNE plot according to cell-types. (B) The replicates combined. (C) Clustering

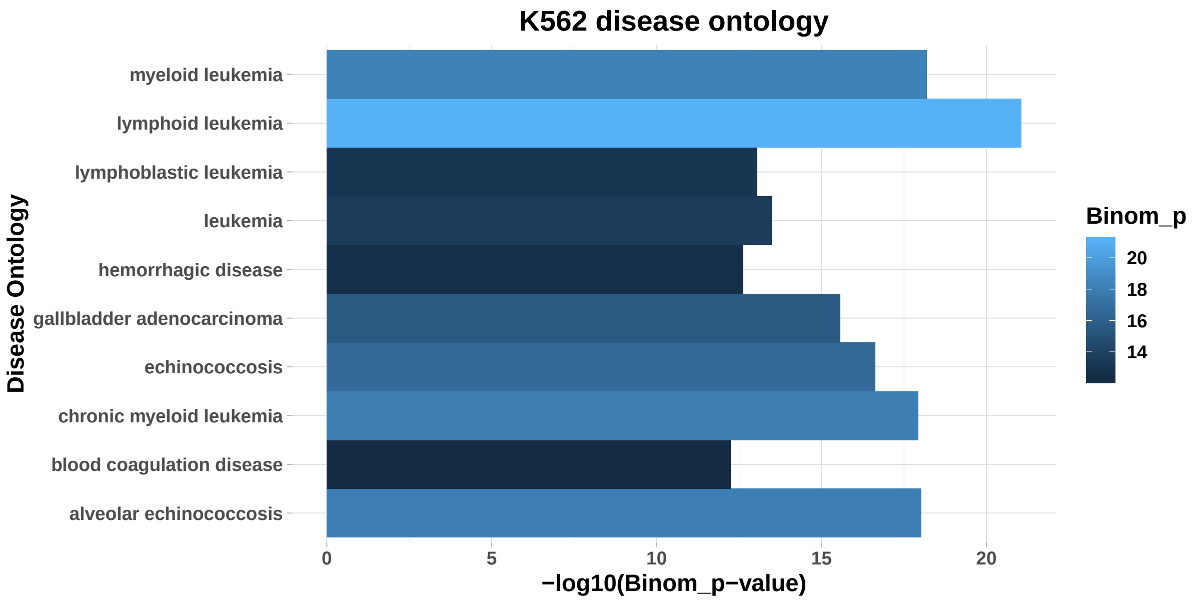

FIG. S5 Disease ontology of K562 cell line. The two topmost disease ontology based on statistical significance is myloid leukemia and lymphoid leukemia which is what this cell line is.

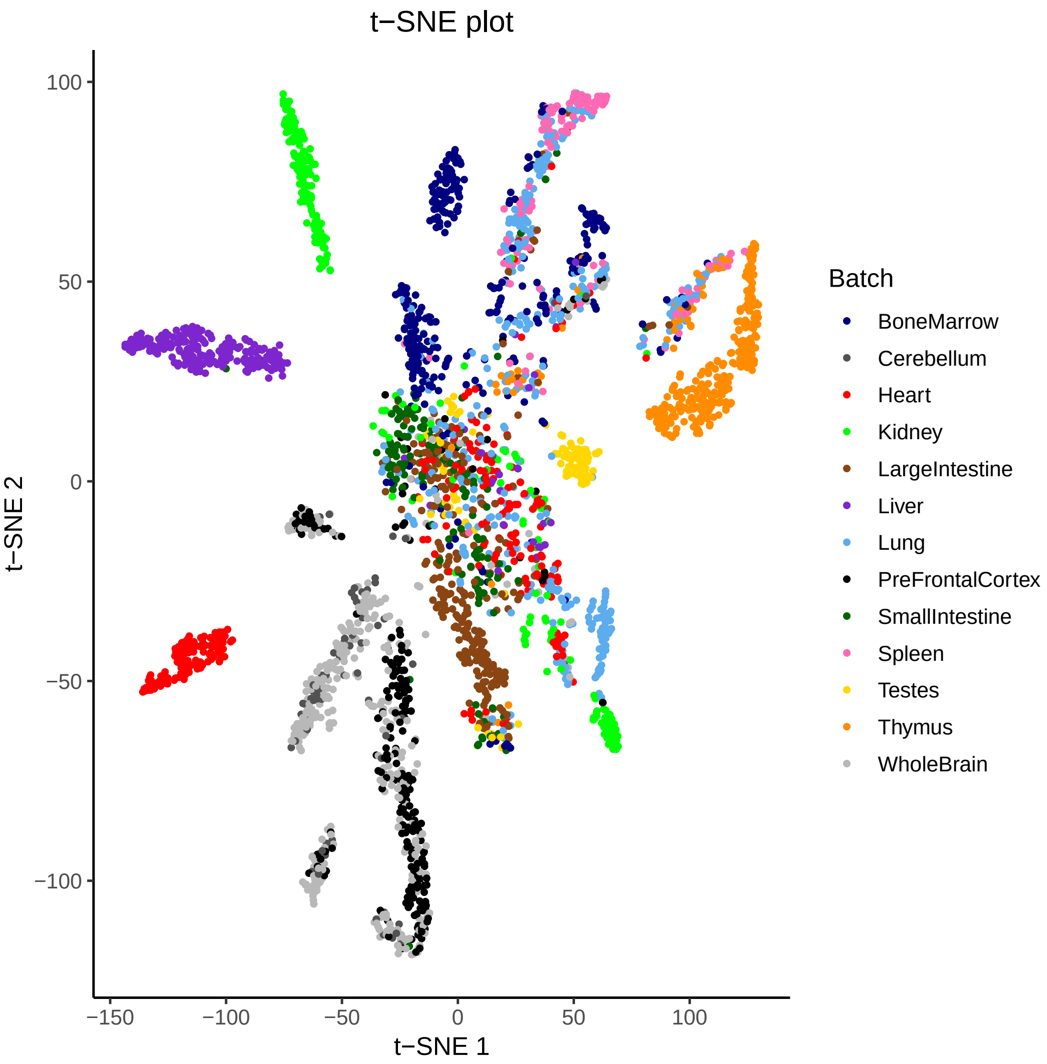

FIG. S6 t-SNE plot with 3000 randomly selected cells from Cusanovich et. al. single-cell Atlas of In Vivo Mammalian Chromatin Accessibility
